# FLUX (Fluid Intelligence Luxembourg): Development and Validation of a Fair Tablet-Based Test of Cognitive Ability in Multicultural and Multilingual Children

**DOI:** 10.3390/jintelligence13110139

**Published:** 2025-11-03

**Authors:** Dzenita Kijamet, Rachel Wollschläger, Ulrich Keller, Sonja Ugen

**Affiliations:** Luxembourg Centre for Educational Testing, Faculty of Humanities, Education and Social Sciences, University of Luxembourg, Maison des Sciences Humaines, 2, Avenue de l’Université, L-4365 Esch-sur-Alzette, Luxembourg; rachel.wollschlaeger@uni.lu (R.W.); ulrich.keller@uni.lu (U.K.); sonja.ugen@uni.lu (S.U.)

**Keywords:** language-fair assessment, nonverbal test, multilingual context, general cognitive ability, elementary school

## Abstract

Nonverbal tests assess cognitive ability in multicultural and multilingual children, but language-based instructions disadvantage non-proficient children. This is a growing concern worldwide due to the increasing number of multilingual classrooms. The tablet-based FLUX (Fluid Intelligence Luxembourg) test was developed within a highly multicultural and multilingual educational context to offer not only nonverbal test content but also language-fair animated video instructions. A total of 703 third graders (*M_age_* = 8.85, *SD* = 0.66; 48.8% females, 51.1% males, 0.1% with no gender specified) were included in the standardisation sample and were assessed with tasks measuring figural fluid intelligence, quantitative fluid intelligence, visual processing and short-term memory. The test proved sufficiently reliable (FLUX Full-scale: McDonald’s Omega = 0.94; split-half = 0.95). Test fairness was ensured by analysing each item for Differential Item Functioning (DIF) on children’s background characteristics (language spoken at home, socioeconomic status, gender). Its factorial structure was confirmed using Confirmatory Factor Analysis (CFA). Further validity evidence was provided by determining its concurrent and criterion-related validity (correlations with a test of cognitive ability and educational achievement scores). Research implications and future prospects in promoting equal opportunities in a heterogeneous multilingual educational context are discussed.

## 1. Introduction

Despite a long history of research and debate, there is still no consensus between the theoretical and practical aspects of intelligence (Canivez and Youngstrom 2019). While cognitive characteristics are viewed in Western countries as important for defining an ‘intelligent child’, other countries value, for instance, motivation or social skills more (Neisser et al. 1996). Among various attempts to define intelligence, two approaches have remained particularly influential to date. The psychometric approach, initiated by Charles Spearman, sought to understand the theoretical structure of intelligence (Spearman 1904), whereas the pragmatic approach introduced by Alfred Binet aimed to create a norm-referenced measure to identify children in need of special education (Binet and Simon 1905). Alongside the challenge of defining intelligence, concerns about cultural and/or linguistic bias in intelligence testing have persisted since the introduction of Binet’s first intelligence scale. As non-native children were not included in its normative sample (Terman 1916) but were later tested with such a language-loaded test, they became overrepresented in special education classes (McCallum 2017), which subsequently led to legal disputes and legislative action (Reynolds and Suzuki 2013).

In spite of these persistent challenges, test development continued over the following decades, drawing on empirically validated theoretical models (e.g., Three-Stratum theory: Carroll 1993; Gf-Gc theory: Cattell 1963; Cattell-Horn-Carroll theory: McGrew 2005). This has led to the widespread finding that intelligence (widely defined as general cognitive ability; Haier 2017) is one of the most reliable indicators of academic and professional success (Kuncel et al. 2004; Neisser et al. 1996; Roth et al. 2015). In fact, it is the strongest predictor, explaining more variance than any other predictor (e.g., Neisser et al. 1996; Roth et al. 2015). The use of (general) cognitive ability tests in children has been widely demonstrated in educational counselling and interventions (e.g., identification of underachievement or specific learning disorders [SLD]) (Baudson and Preckel 2013). However, to be reliable and valid, these tests must be conducted in the language in which the child is proficient. In fact, language-loaded tests may lead to test bias in the form of construct-irrelevant variance. Rather than assessing the intended construct (cognitive ability), they become a language test (Hassett et al. 2024). For children affected by construct-irrelevant variance, the use of nonverbal assessment can provide a more accurate measure of their cognitive abilities (Hassett et al. 2024).

Given the global increase in multicultural and multilingual classrooms due to global migration and mobility, this represents a significant challenge worldwide (Parveen et al. 2022). Consequently, there will remain high demand for nonverbal tests, especially for cognitive assessment (Hassett et al. 2024).

Awareness about the impact of culture and/or language on test performance has existed since the early 1900s (Schaap 2011). Nonverbal assessments began to advance notably during the First World War, when the armed forces needed to evaluate the cognitive abilities of illiterate or foreign-born military recruits (Hassett et al. 2024; McCallum 2017). Unlike the language-loaded Army Alpha form, these groups were assessed with the Army Beta form, a group-administered nonverbal test that included tasks such as picture completion, digit symbol, and picture arrangement. In addition, psychologists developed the Army Individual Performance Scale, an individually administered nonverbal test designed to detect malingerers, which involved manipulating concrete materials (e.g., cube construction and object assembly). Both nonverbal tests—group and individual—greatly influenced Wechsler’s later development of the Performance Scale (Hassett et al. 2024; McCallum 2017). Since then, further nonverbal tests were developed to provide more culture/language-fair assessments of cognitive ability (e.g., CFIT: Cattell 1949; Nonverbal Scales of the K-ABC; Kaufman and Kaufman 1983; NNAT: Naglieri 1996).

While most of these tests have nonverbal content (using, for instance, abstract figures), they still have language-loaded instructions. Unfortunately, this puts many children who are not proficient in the test language at a disadvantage (Ugen et al. 2021), as relying on language-specific diagnostic tools can produce biassed and invalid test results (Ortiz and Dynda 2005).

For nonverbal assessments to be used effectively, verbal instructions either should ideally not be required or should be minimised as much as possible (Hassett et al. 2024; Oakland 2009). To achieve this, some existing nonverbal tests use pantomime, gestures, or static pictorial instructions to reduce linguistic demands (for further details and examples, see Section 2.1.2). However, such types of nonverbal communication can be difficult to understand and may introduce cultural bias, as the interpretation of gestures can vary across cultural groups (Archer 1997).

Additionally, some of these tests may still contain pictorial contents that are more prevalent in certain cultures than others, making them culturally biassed (McCallum 2017) since children who might be unfamiliar with the culture are expected to have culture-specific knowledge (Salvia et al. 2012).

This study aims to fill this gap by developing a tablet-based test battery called FLUX (Fluid Intelligence Luxembourg), tailored to the specific needs of multilingual educational settings. Using not only nonverbal/culture-fair test contents but also language-fair instructions presented through child-adapted animated videos instead of static pictorial instructions, FLUX is conceived to measure the general fluid cognitive ability of third-grade children in a fair manner, regardless of their cultural or linguistic background.

In addition to children’s cultural and linguistic backgrounds, we also considered their socioeconomic status (SES) and gender when studying their performance. In fact, it has been found that children from low SES backgrounds generally score lower on cognitive ability tests compared to their high SES peers (Bradley and Corwyn 2002; Schoon et al. 2012; Strenze 2007). When it comes to considering gender differences in cognitive ability, there is a controversial debate. While some studies have reported no differences in cognitive ability between males and females in primary schoolchildren and adolescents (e.g., Camarata and Woodcock 2006; Giofrè et al. 2022; Goldbeck et al. 2010; Keith et al. 2011; Pezzuti and Orsini 2016), some have highlighted domain-specific variations. For instance, Giofrè et al. (2022) found in their meta-analysis of WISC (Wechsler Intelligence Scale for Children) batteries no differences in tasks measuring fluid cognitive ability but observed that females performed better on processing speed subtests (requiring writing symbols), whereas males showed an advantage on certain visuospatial subtests (e.g., block design). In contrast, other studies have found that females tend to have higher general cognitive ability scores (e.g., Calvin et al. 2010; Härnqvist 1997; Reynolds et al. 2008).

Finally, we aimed for an economic assessment of children’s general fluid cognitive ability level, which is why the FLUX test is normed as a group test (e.g., Ford and Dahinten 2005), while children could still work on the tasks in their own rhythm as the test is tablet-based.

As the development of a robust standardised test should be grounded in empirically validated theoretical models, it is also important to consider the development of cognitive abilities in children. The following will therefore outline the rationale for assessing children’s general fluid cognitive ability.

### 1.1. Development of Cognitive Abilities in Children

In intelligence research, one empirical phenomenon is well established: different cognitive abilities are correlated with one another, also known as a positive manifold (Spearman 1904). In light of this phenomenon, it is widely accepted that there is an underlying factor of general cognitive ability, commonly referred to as *g* (Carroll 1993; Gottfredson 1997; Jensen 1998; Thorndike 1994), which is responsible for interindividual differences in cognitive ability (Jensen 1998). According to Cattell (1963), *g* is composed of two distinct abilities: fluid intelligence (*Gf*) and crystallised intelligence (*Gc*). In his investment theory, Cattell (1963) stated that *Gf* is an innate cognitive ability independent of cultural factors. It involves abstract reasoning abilities necessary for adapting to novel and complex situations (Heitz et al. 2004). During childhood and adolescence, *Gf* helps acquire skills and knowledge through learning that contribute to the formation of *Gc* (knowledge gained via parental or school education) (Cattell 1963; Schweizer and Koch 2002). Hence, *Gc* is shaped by cultural factors and continues to develop through the accumulation of knowledge with age. On the other hand, *Gf* begins to decline in early or middle adulthood (Rose and Fischer 2011). Evidence for age-related decline in *Gf* is also largely supported by ageing research using well-known standardised cognitive ability tests (for more details, please refer to, e.g., Kaufman et al. 2008, 2016; Salthouse 1998, 2005).

Due to their young age, children have had fewer opportunities to develop their *Gc* entirely. Also, “schools tend to standardise the growth of the knowledge base during the time of schooling” (Schweizer and Koch 2002, p. 78). Moreover, cognitive ability primarily depends on the current state of brain development and the respective neural processes observed through *Gf* (Cattell 1987). Hence, *Gf* is considered the most accurate measure of children’s *g* (Baudson and Preckel 2013; Cattell 1963; Schweizer and Koch 2002).

*Gf-Gc* and *g* factor theories are now encompassed in the hierarchical Cattell-Horn-Carroll (CHC) model of cognitive abilities (McGrew 2005), the most comprehensive and empirically supported psychometric theory of the structure of cognitive abilities. Validity evidence for the CHC model is supported by not only factor-analytic but also non-factor-analytic research (including, e.g., heritability, neurocognitive, developmental, educational, and occupational outcome prediction studies) (Horn and Blankson 2005; Horn and Noll 1997). It comprises three strata: *g* at the highest level (stratum III), followed by broad abilities at the second (stratum II) level, and narrow abilities at the third (stratum I) level. Not being a static theory, the CHC model has been evolving since its establishment (Flanagan and Dixon 2014). According to Schneider and McGrew (2018), CHC broad abilities can be categorised into six groups: domain-general reasoning capacity, domain-specific sensory abilities, memory and efficiency, motor abilities, speed efficiency, and acquired knowledge capacities. As mentioned above, the latter (acquired knowledge abilities) are not considered the most accurate measures of children’s *g*. As the CHC model of cognitive abilities is widely used for selecting and developing tests of cognitive abilities (Alfonso et al. 2005; Reynolds et al. 2014), cognitive abilities that do not measure children’s *g* accurately can be excluded from the model and assessment.

Concerning “memory and efficiency” (Schneider and McGrew 2018), research shows that *Gf* involves the same brain systems related to *Gswm* (short-term memory [*Gsm*] and working memory [*Gwm*]), and attentional control (e.g., Burgess et al. 2011; Duncan et al. 2000; Gray et al. 2003; Kocevar et al. 2019), whereas *Gc* involves brain systems related to long-term memory (learning efficiency and retrieval fluency) (e.g., Geary 2005; Kocevar et al. 2019; Tamnes et al. 2010).

Furthermore, a study by Hornung et al. (2011) found that *Gsm*, *Gwm*, and *Gf* are highly correlated in children (5–7 years old), and the *Gwm-Gf* relationship is primarily influenced by the *Gsm* storage, suggesting that *Gsm* measures are as reliable as *Gwm* measures when it comes to evaluating children’s core storage capacity, which is essential for reasoning and problem-solving abilities.

In the context of domain-specific sensory abilities, when *Gv* (visual processing) is assessed through tasks that entail mentally transforming figures, it has been shown to be closely related to *Gf* (Buckley et al. 2018; Carroll 1993). Further details are provided in Section 1.3.

Finally, regarding abilities related to speed, they are considered to be not the best predictors of cognitive performance in children under the age of 10 (Lavergne and Vigneau 1997).

While it is important—when assessing children—to only include cognitive abilities that measure children’s *g* as accurately as possible, it is equally important to consider adapting the CHC model when evaluating cognitive ability in a highly multicultural and multilingual educational context. But first, the rationale behind the need for developing such a new test for multicultural and multilingual children will be explored in more detail.

### 1.2. Rationale for Developing a New Test for Multicultural and Multilingual Children

Given the global increase in cultural and linguistic diversity within classrooms, large-scale studies (such as Programme for International Student Assessment [PISA] and Progress in International Reading Literacy Study [PIRLS] have consistently confirmed that many educational systems, struggle to provide equal educational opportunities for many children from diverse social, cultural, and ethnic backgrounds (e.g., Martin et al. 2015; OECD 2018). One of the biggest challenges is related to language, in educational contexts in which the language spoken at home differs from the instruction language (e.g., Martin et al. 2015; OECD 2018). Children who speak the instruction language at home (native children) consistently outperform those who do not (non-native children) in scholastic tests, which might be due to difficulties with the test language rather than limited learning potential (e.g., Greisen et al. 2021; Méndez et al. 2019). By assessing a child’s cognitive ability independent of school content (as far as possible), the newly developed language-fair FLUX test, which employs nonverbal test content and language-fair animated video instructions, could be used to identify these children—so-called underachievers—at an early stage and provide them with the support they need to succeed in school. On the other hand, it could also be used to identify overachievers—children whose school performance exceeds their cognitive potential (Dings and Spinath 2021)—and to diagnose intellectual giftedness (high cognitive potential) or the opposite intellectual disability (low cognitive potential) (e.g., Campbell et al. 2021).

In terms of differential diagnostics of a specific learning disorder (SLD), alongside literacy or mathematical tests, a test of cognitive ability might be useful to exclude an intellectual disability (Dilling et al. 2015). In this context, early assessment of a child’s cognitive ability is crucial to support the diagnosis, which can typically be made no earlier than after two years of formal education (Ugen et al. 2021). Moreover, it can be used to identify weaknesses in some cognitive abilities present in children with SLD that are crucial to learning (such as *Gsm* and *Gwm*) (e.g., Giofrè et al. 2016; Peng and Fuchs 2014). Likewise, in this case, the language should not impact the results to ensure accuracy and assist practitioners in guiding interventions effectively.

To develop and validate a language-fair test that accurately captures children’s cognitive potential, a heterogeneous multilingual educational context at the third-grade level was essential. Luxembourg provides an excellent example of a highly multicultural and multilingual educational context (for further details, see Section 1.4 Research Questions of the Present Study) that strives to minimise the impact of culture and language on a child’s test performance.

### 1.3. Adapting the CHC Model to a Culture and Language-Fair Assessment Context

Wilhoit (2017) suggested that the CHC model can be used to assess cognitive abilities in individuals with limited language proficiency by selecting tasks that are not language-loaded.

Furthermore, as recommended by experts (e.g., Coleman et al. 1993; Daseking and Petermann 2015; Joél 2018; Schneider and McGrew 2018; Wilhoit 2017), measures requiring cultural and school-taught knowledge should be excluded when assessing multilingual children to account for possible differences in the cultural backgrounds associated with their specific language profiles.

For motor and some domain-specific sensory abilities (e.g., auditory ability), there is insufficient evidence to include them within the CHC model (Schneider and McGrew 2018). One of the sensory abilities for which there are reliable and valid measurements is visual processing (*Gv*) (Schneider and McGrew 2018). Hopkins et al. (2019) discovered a significant relationship between *Gv* and academic performance in reading and mathematics for second-grade students. *Gv* can be assessed nonverbally, and generally, nonverbal tests of cognitive abilities draw heavily on *Gv* and *Gf* (DeThorne and Schaefer 2004). *Gf* is crucial for learning (Kyllonen and Kell 2017) and predicts scholastic performance (Finn et al. 2014; Postlethwaite 2011). Coleman et al. (1993) emphasised that nonverbal cognitive ability tests should measure complex reasoning (*Gf*) and require flexibility in reasoning strategies. According to Guttman’s (1965) radex model with *g* at its centre, abilities that require complex reasoning, such as figural reasoning or quantitative reasoning, are closer to the centre of the model (Lohman 1993; Marshalek et al. 1983; Tucker-Drob and Salthouse 2009).

Finally, experts of culture-fair assessment advised against timed procedures for assessing children with different cultural backgrounds, as some cultures value more precision than quick decision-making (Coleman et al. 1993; Jensen 1980; McCallum and Bracken 2018). Hence, power tests (that measure a child’s knowledge) are considered to be more culturally fair than speed tests (that measure a child’s ability to answer correctly within a set time limit) (Kim and Zabelina 2015).

**Figure 1:**
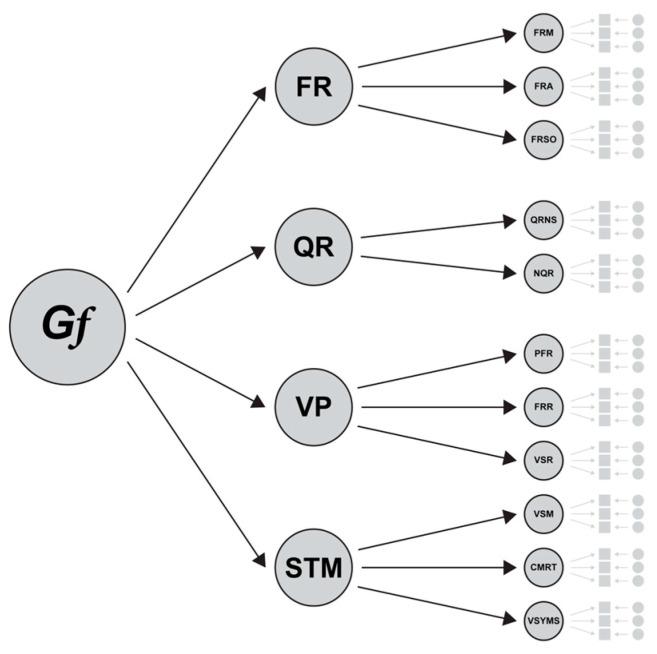
FLUX model: Adapted CHC framework for multicultural and multilingual children with *Gf* (general fluid cognitive ability) at the apex, followed by four broad cognitive abilities (cognitive domains), each assessed through a specific set of narrow abilities (subtests). FR: Figural fluid reasoning (FR), QR: Quantitative fluid reasoning (QR), VP: Visual Processing (VP), STM: Short-term memory (STM).

According to this rationale, FLUX is based on an adapted CHC model for culture and language-fair assessment, incorporating general fluid cognitive ability (*Gf*) at the apex followed by these four cognitive domains: (1) Figural fluid reasoning (FR), (2) Quantitative fluid reasoning (QR), (3) Visual Processing (*Gv*; which we named VP), and (4) Short-term memory (*Gsm*; which we named STM) (see Figure 1; Section 2.4.1. provides a detailed overview of the four cognitive domains and their corresponding subtests). This leads to a more comprehensive multidimensional assessment of a child’s *Gf* level, which is also considered fairer than a unidimensional assessment (e.g., only applying progressive matrices to assess *Gf* in children), as the latter is more appropriate for low-stakes screening assessments (McCallum 2017; McCallum and Bracken 2018). Providing assessments of multiple constructs allows us to create a child’s profile, identifying their strengths and weaknesses (Wilhoit 2017). This is particularly useful in, for instance, high-stakes assessment or a differential diagnostic setting (Franklin 2017).

To ensure culture/language-fair assessment of the four cognitive domains, it was also essential to adhere to established guidelines for developing culture/language-fair items and instructions (details of this more methodological process are provided in Section 2.1).

### 1.4. Research Questions of the Present Study

FLUX was developed within Luxembourg’s trilingual primary school system. Luxembourg’s educational system is unique in that it incorporates multiple languages into its curriculum. Luxembourgish is the primary language taught in kindergarten, which switches to German as the language of instruction for reading, writing, and mathematics (MENJE 2020). Both languages are linguistically similar (i.e., as close as a German dialect to standard German; Martini 2021). Notably, only about 5% of children speak German at home, yet their listening comprehension in both languages does not differ significantly from that of their peers who speak Luxembourgish at home (Hornung et al. 2023).

French is introduced as a foreign language from the first grade onwards (MENFP 2011). Approximately one-third of the children in Luxembourg speak Luxembourgish at home, and the remaining two-thirds (referred to as non-native speakers) speak other languages as their first language at home (MENJE and SCRIPT 2022) (e.g., Portuguese, Italian, Spanish, French, South Slavic, English).

Tailored to the specific needs of multicultural and multilingual educational settings, FLUX is conceived to be a psychometrically sound measure of a child’s *Gf* and provides insight into third graders’ learning potential. To be a psychometrically sound measure, it should be both reliable and valid (AERA et al. 2014).

The present study endeavours to examine FLUX’s three fundamental psychometric properties, which are:

(1) To ensure whether FLUX measures what it is designed to measure (a child’s general fluid cognitive ability). This involves investigating (a) if its hypothesised factorial structure is supported by empirical data (by applying Confirmatory factor Analysis; CFA), and (b) determining its concurrent and criterion-related validity by correlating it with a test measuring cognitive ability (same construct), and with educational achievement measures: in mathematics for convergent validity (related constructs), and German reading and listening for divergent validity (unrelated constructs).
(2) To explore the reliability of FLUX by investigating its internal consistency (applying McDonald’s Omega, ω, and split-half reliability) by examining if a group of items reflects the same underlying construct.
(3) To determine whether FLUX is assessing the *Gf* of third-grade children in a fair manner, independent of their background characteristics (SES, language spoken at home, and gender) (by applying DIF, to test for measurement invariance at the item level).

## 2. Materials and Methods

### 2.1. Test Development: Item and Instruction Development

#### 2.1.1. Item Development

Items were developed based on theoretical and empirical research (including the pre-test explained below) conducted on culture/language-fair assessment. In addition, child-friendly task contents and colour combinations taking colourblindness into account were chosen, in collaboration with experts in vision who reviewed all testing materials.

In Jensen’s guidelines (1980) for culture/language-fair measures of cognitive ability, novel abstract content that requires complex reasoning and problem-solving is recommended instead of pictures or reading passages. Such abstract content is usually equally novel to all test takers, minimising the potential impact of *Gc* (Johnsen 2017). Following this guideline, most items were constructed from simple abstract figures, excluding linguistic content and unnecessary visual elements or pictures that could introduce culture/language bias, increase visual load, or distract children. Moreover, any material that might evoke emotional distress was avoided throughout the test.

In addition, training/practice items are recommended to ensure that all children are familiarised with the task before actual assessment (e.g., Coleman et al. 1993; Jensen 1980).

These recommendations contribute to creating fair conditions for assessing the cognitive abilities of children as equally as possible, as advocated by the American Educational Research Association, American Psychological Association, and National Council on Measurement in Education (AERA et al. 2014).

Additionally, neither expressive nor receptive language should have an impact on the test results (Bracken and McCallum 2016). Hence, the response mode should not require speaking or writing. Multiple-choice and drag-and-drop formats are used on tablets to enable children to respond.

Based on these recommendations, a pool of 445 items was first created for a piloting phase involving 25 children. During this phase, structured interviews were conducted to collect qualitative feedback on children’s task perceptions, problem-solving techniques, and understanding of the first animated instructional videos. In addition, expert evaluations from teachers, psychologists, and experts in vision focused on age appropriateness, cognitive demands, and visual design and accessibility. Drawing on these insights, the item pool was refined to 371 items for the pre-test. During the pre-test, approximately 200 children were tested to assess the psychometric properties of each item (e.g., discrimination, difficulty level—to order them by increasing difficulty) with item response theory (IRT, using the TAM package; Robitzsch et al. 2022), which is also preferred for power tests (De Ayala 2009). Before administering the test on a tablet, a paper-and-pencil version was used for the pre-test. As part of the pre-test, a master’s student analysed performance differences between paper-and-pencil and tablet assessments in a multilingual context with 55 children. In line with Hamhuis et al. (2020), no differences in performance between the two modes were found. Hamhuis et al. (2020) examined the performance of 532 fourth-grade children in mathematics and science using tablet and paper-and-pencil assessments and found no significant differences in student abilities related to the test mode.

After IRT, the remaining items were subjected to Differential Item Functioning (DIF, using the TAM package; Robitzsch et al. 2022) to ensure fairness by testing whether the items work comparably in children with different backgrounds (SES, language spoken at home, gender) (AERA et al. 2014; Banerjee and Papageorgiou 2016; Messick 1989). Following the data analysis (IRT and DIF), some items were removed, others were modified, while the majority remained unchanged for nationwide standardisation (main-test) using a representative sample of multicultural and multilingual schoolchildren from Luxembourg. In line with the item analysis strategy sketched above, main-test data were analysed, and the number of test items was ultimately reduced to 132 items to ensure FLUX’s psychometric quality and to facilitate practical assessment.

#### 2.1.2. Instruction Development

In some existing nonverbal tests, pantomime and gestures have been used to minimise the language in test instructions (e.g., UNIT: Bracken and McCallum 1998; CTONI-2: Hammill et al. 2009; SON-R 2-8: Tellegen et al. 2018). However, this kind of nonverbal communication can be unfair since not everyone understands it, and the cultural meanings behind gestures can vary (Archer 1997).

The Wechsler Nonverbal Scale of Ability (WNV; Wechsler and Naglieri 2006) includes static pictorial instructions to help children understand what is required of them. Yet, according to a meta-analysis conducted by Höffler and Leutner (2007), dynamic visualisations (animations) are more effective in aiding examinees to understand instructions than static pictures. In addition, Lakin (2010) discovered that incorporating additional video animation into simple verbal instruction in English and Spanish significantly improves the performance of second graders on a figure analogy test.

However, Greisen et al. (2018) showed that using touch-screen tablets with animation-based instructions was more effective than verbal instructions for first graders to understand mathematical tasks. Therefore, FLUX used animated videos with language-fair instructions that were pretested for comprehension before applying them in the main test.

Regarding the psychometric implications of touch screen tablets, Pitchford and Outhwaite (2016) conducted a study on assessing cognitive (spatial ability, visual attention, short-term and working memory) and motor (manual processing speed, manual coordination) abilities in elementary schoolchildren (*M* = 97.15 months) and found that touch screen tablet technology can provide reliable and valid psychometric measures of performance in early years. Semmelmann et al. (2016) discovered that children as young as two years old can effectively interact with tablets, comprehend the tasks assigned, and provide answers through the device. These findings suggest that tablet technology has the potential to be used in cross-cultural comparisons and research.

### 2.2. Participants

The representative sample was collected from February 2023 through June 2023. From among 714 children, results are reported for the overall 703 third-grade children (*M* = 8.85 years; *SD* = 0.66), as children diagnosed with an SLD (eight children), a mental disorder (one child) or those who chose not to continue the test (two children) were omitted. Depending on whether children were absent on the first or second testing day, the number of participants may vary between subtests (see Table A1).

All children from thirty-five public schools across all fifteen school districts in Luxembourg were assessed in their regular classrooms. Children’s background characteristics were assessed with a student and parent questionnaire. Detailed information on children’s background characteristics is provided in Table 1. Children were divided into two language groups. Native speakers comprised children speaking Luxembourgish and/or German with both or at least with one parent at home. Non-native speakers comprised children speaking other languages at home.

Children’s SES was assessed by the parent questionnaire using the International Socio-Economic Index of Occupational Status (ISEI) (see Section 2.4.2). Information on the highest parental ISEI (HISEI) was available for 568 children, as some parents did not fully complete the questionnaire. The sample comprised children from a wide range of socioeconomic backgrounds (with a HISEI of 16.25 to 69.90), which is reflective of the Luxembourgish school population (see Hornung et al. 2014).

### 2.3. Procedure

In the presence of the teacher, trained test administrators conducted the tablet-based group test for children in their quiet classrooms using standardised guidelines. Prior to testing, teachers were provided with information letters for legal representatives explaining the aim of the project and the confidential data handling procedures. Legal representatives were informed that they may opt out of the study by contacting the child’s teacher.

The study was conducted following the research ethics guidelines of the University of Luxembourg and approved by its ethics review panel (ERP 22-026A).

Due to the total test duration, the administration was split into two testing sessions, each starting in the morning to maintain standardisation and decrease the possibility of cognitive fatigue caused by later points in time (Sievertsen et al. 2016). Each testing session lasted about two school hours, including breaks. Subtests and items were administered in a fixed order established during the pre-test phase, as it is recommended for standardised tests. Adherence to this order was critical for ensuring standardised administration, as any deviations could compromise the test’s psychometric properties (AERA et al. 2014). Children were seated in their exam constellation, facing the blackboard and screen. The test was run via the online test and assessment system OASYS (Sonnleitner et al. 2024). Before the actual test and always during the first session, an introduction to the class was provided in Luxembourgish (the communication language used at school), explaining the purpose of the test, the testing schedule, and a brief instruction on how to use tablets. Additionally, children were guided on how to enter student codes and passwords. Each password, composed of two letters followed by one to two numbers (e.g., “ak1”), was written on the blackboard before the start of each subtest.

Language-fair instruction videos were displayed on the screen to introduce the subtest. A fox demonstrates the task to the children using body language (such as smiling and pointing) and language-fair speech sounds (such as mumbling and pondering). During the explanation, the fox intentionally selects a wrong answer to show the feedback children would receive (a red smiley with the corners of the mouth down) if they chose the wrong answer during training. Then, the correct answer is justified and selected (a green smiley with the corners of the mouth up appears). During the training phase, children were given feedback in the form of a red smiley or a green smiley, indicating whether they understood the task or not. The only selectable option is the green smiley, so they could only proceed if they solved the training/practice items correctly by pressing on the smiley. The items are designed to be intuitive, and with the help of feedback and instructions, children can understand them easily. However, if they had any doubts, they could ask questions to clarify. Once they understood the task and it was ensured that all children were equally familiar with the requirements, they could move on to the test phase by pressing a play button. During this phase, they were not allowed to ask questions or receive feedback on their solutions. Each child worked individually at their own pace, with sufficient time to complete each item. Progression to the next item was only possible after an answer had been selected.

### 2.4. Measures

#### 2.4.1. FLUX

Based on the adapted CHC model, FLUX aims to assess children’s *Gf* in a fair manner. The following describes its structure in more detail to present the whole test. Table A1 (see Appendix A) provides information on the number of items for each subtest. Since *Gf* is the most accurate measure of children’s *g* (Baudson and Preckel 2013; Cattell 1963; Schweizer and Koch 2002), the first two domains were constructed to require inductive and deductive reasoning essential to understanding and solving novel problems (e.g., Kane and Gray 2005; Primi 2002; Swanson et al. 2008).

The first domain is **Figural fluid reasoning (FR).** Due to the novelty of abstract figural reasoning and its location near the centre of Guttman’s radex model (Marshalek et al. 1983; Tucker-Drob and Salthouse 2009), which provides a better representation of Cattell’s Gf (Lohman 1993), we chose the following three subtests:

- ***Figural Reasoning—Matrices (FRM).*** To find the missing figure, children were required to decipher the connection between four to nine abstract figures connected per row, per column, diagonally or in several directions, by selecting the correct answer from four possible solutions.
- ***Figural Reasoning—Analogies (FRA).*** The task involved two rows of abstract figures positioned in relation to each other. Children had to identify the rule based on the first row (e.g., big becomes small) to complete the equation on the second row, where the figure on the right side was always missing, by selecting the correct answer from four possible solutions.
- ***Figural Reasoning—Sequential Order (FRSO).*** Children had to correctly complete the respective sequences of four abstract figures. Starting from the initial figure, they had to find out what happened to it (e.g., the figure becomes smaller and smaller) and then complete the sequence by selecting the correct one from four possible solutions.

The second domain is **Quantitative fluid reasoning (QR).** QR involves complex reasoning using induction or deduction in terms of numbers, quantitative relations, and operators without requiring advanced quantitative (mathematical) knowledge (Schneider and McGrew 2018). Thus, to measure complex reasoning (*Gf*), the task conceptualisations should be simple and accessible to third-grade children while requiring reasoning, making the following two subtests complex enough to assess QR:

- ***Quantitative Reasoning—Numerical Series (QRNS).*** Children were presented with sequences of five to six numbers with one number (the second last) missing in each sequence. By using an operation (i.e., addition, subtraction, multiplication, or division), they were able to infer the rule applicable (e.g., +1, +1 or −2, −1) to the series, which allowed the missing number to be deduced by selecting the right one among four answer possibilities.
- ***Non-Symbolic Quantitative Reasoning (NQR).*** Children were shown a 3 × 3 grid with dots or bars in each cell except one, which was empty. By first inferring that two identical colours represent addition (e.g., white-white or black-black) and two different colours (e.g., black-white) represent subtraction, children were able to determine the quantitative relationship between figures in each row and column by applying the right operation and choosing the right answer from four possible answers.

The third domain is **Visual Processing (VP).** VP refers to the ability to solve visual-spatial problems using simulated mental imagery (along with currently perceived visual information) (Schneider and McGrew 2018). Visual measures that require mental transformation of figures (by, e.g., rotating them, changing their shape, or mirroring them) after visually perceiving them are considered to be related to *Gf* (Buckley et al. 2018; Carroll 1993). Hence, the following three subtests have been developed to assess VP accurately:

- ***Paper Folding Reasoning (PFR).*** A drawing of a sheet of paper on top of the screen had been folded either once (from top to bottom) or twice (from top to bottom and right to left). Additionally, each paper had one or more holes cut out of it. The task required children to visualise the paper being unfolded and predict its appearance (by selecting an answer among four) while accounting for the holes.
- ***Figural Rotation Reasoning (FRR).*** Children were presented with a figure on the top of the screen and required to find the exact figure in a rotated form from four options below using mental rotation. It is important to note that the upper figure must not be imagined as a mirror image, and children were not allowed to rotate the tablet manually while solving the task.
- ***Visual Spatial Reasoning (VSR).*** This task required children to mentally connect three puzzle pieces and rotate them mentally if needed to create the corresponding figure at the top. To respond, children had to select three out of six possible answers.

Lastly, the fourth domain is **Short-term memory (STM).** STM refers to the ability to hold a limited amount of information for a limited period of time (seconds) (Baddeley 2000). Using simple memory span tasks is recommended when assessing STM, and if visual memory tasks are presented in a sequence, they are more STM than VP measures (Schneider and McGrew 2018). Thus, the following memory tasks were developed to assess STM:

- ***Visual-Spatial Memory (VSM).*** In a 4 × 4 grid, a sequence of three to seven apples appeared simultaneously in their respective cells. Children were asked to memorise the position of each apple and reproduce it by selecting the corresponding cells in an empty grid once the apples disappeared. They could only move on to the next step once they had reproduced the quantity of apples shown previously (if two apples were projected, children had to select two cells to be able to move to the next item).
- ***Counting-Memory-Recall Task (CMRT).*** A sequence of yellow squares with dots appeared on the screen; each square displayed a certain number of small quantities of green dots (minimum one dot, maximum five dots). With an innate ability to subitise up to about four dots without counting, children can determine the exact number of dots on each square in a sequence even without counting knowledge (Davis and Pérusse 1988; Kaufmann et al. 1949), enabling them to determine the number of dots quickly and accurately. The task started with sequences of three squares of dots and progressed to sequences of six squares of dots. During each presentation, children were required to memorise the respective sequence, and as soon as it disappeared, they had to drag and drop the squares of dots on the lower screen into empty sequenced boxes in the answer format on the upper screen to reproduce the recently shown sequence in the correct order.
- ***Visual Symbolic Memory Span (VSYMS).*** Abstract figures were presented to the children in a sequence (from two to four). Each figure (trapezoid, circle, triangle) was either yellow or blue and pointed upwards or downwards. The correct sequence of each figure had to be memorised based on its shape, colour, direction, and place. Immediately after the presentation ended, an answer format appeared, and children were asked to reproduce the recently shown sequence by dragging and dropping the figures into empty sequenced boxes.

#### 2.4.2. Measures for Validation

**Shortened version of Raven’s standard progressive matrices (RAVEN-short).** Children were assessed with a shortened Raven version (RAVEN-short) alongside the FLUX assessment to evaluate concurrent validity. This version was developed by Langener et al. (2021) with a machine learning approach that helped to identify the best 15 items for children (9–12 years) and adolescents (13–16 years), respectively.

**Educational Achievement Measures.** To evaluate construct validity, we matched the data (through a procedure ensuring each child’s anonymity) with educational achievement measures. In grade three, educational achievement in mathematics (to evaluate convergent validity), German reading and German listening (to evaluate divergent validity) was obtained through the standardised national school monitoring programme (Épreuves Standardisées[ÉpStan]; Martin et al. 2015) used in Luxembourg to assess whether children have reached the required level of achievement at different stages of their educational curriculum (Martin et al. 2015).

**Background Measures.** Background measures were collected via questionnaires distributed to children (student questionnaire) during the FLUX test and to parents/caregivers as part of ÉpStan.

The student questionnaire collected information on children’s background (including age, gender, the year they started attending Luxembourgish school, and languages spoken at home) and their use of tablets and computers both at home and in the classroom.

The questionnaire for parents/caretakers gathered information on SES variables (e.g., occupation, migration background, and level of education). SES was estimated through the international Socio-Economic index (ISEI) of occupational status (Ganzeboom et al. 1992), taking the highest-rated parent into account (HISEI).

#### 2.4.3. Data Analysis

Answers were coded as correct (1) or incorrect (0). Children had enough time to respond and could only proceed after selecting an answer. As precision was prioritised over quick decision-making, reaction times were not recorded, as power tests are considered to be more culturally fair than speed tests (Kim and Zabelina 2015).

**Figure 2:**
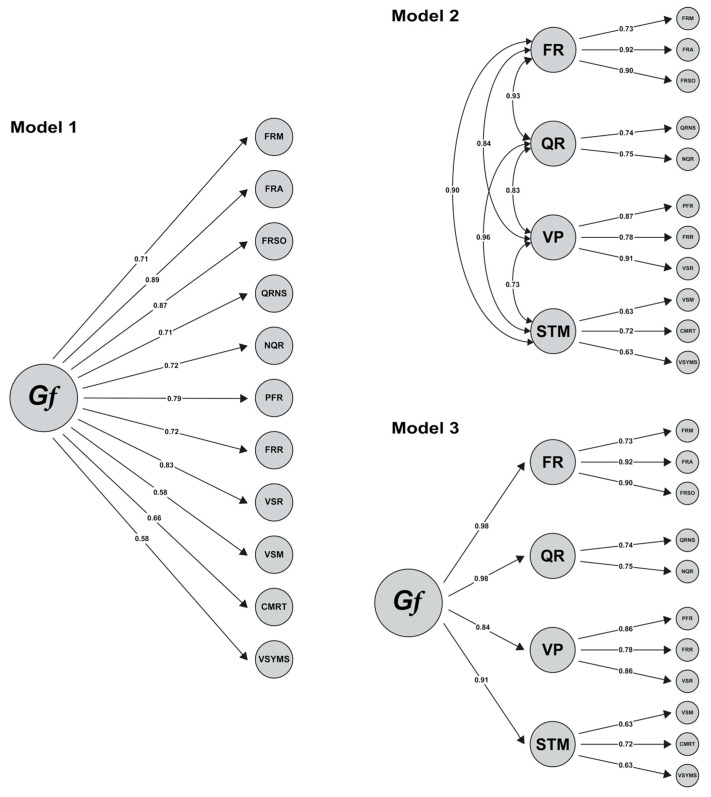
Three Conceptually Different Models of Cognitive Abilities. All items (observable variables) loaded significantly on their relevant core subtest (*p* < .001) but are not displayed due to space limitations. All values in the figure for each model are statistically significant (*p* < .001).

CFA were conducted using the lavaan package (Rosseel 2012) in R software, version 4.3.0 (R Core Team 2023), to determine if empirical data support the theoretical structure. The unidimensionality of each subtest was assessed using CFA (Moosbrugger and Kelava 2020). We applied the diagonally weighted least squares (DWLS) estimator for dichotomous data. The hypothesised model was evaluated using approximate fit indices as follows: Chi-square value to degree of freedom ratio (χ^2^/df) is less than 5 (Schumacker and Lomax 2004), CFI (Comparative Fit Index) and TLI (Tucker–Lewis Index) values being 0.90 and above and RMSEA (Root Mean Square Error of Approximation) and SRMR (Standardised Root Mean Square Residual) values of 0.08 and below (Chen et al. 2008; Gana and Broc 2019; Hu and Bentler 1999). In addition, the hypothesised third-order model was compared to two second-order models (one with all 11 subtests loading on *g* at the apex and one without a *g* at the apex but with subtests loading on their respective domains) (see Figure 2). To conduct this comparison, the chi-square difference test was used (Satorra 2000).

To further evaluate test validity, we investigated concurrent and criterion-related validity obtained by correlating FLUX with RAVEN-short for concurrent validity, with educational achievement in mathematics for convergent validity, and with German reading and German listening for divergent validity. The strength of Pearson correlations was interpreted according to established conventions for effect size interpretation (see Hopkins 2002; Rosenthal 1996). Furthermore, as per the recommendation of Evers et al. (2013), when using two similar instruments to measure the same construct (with data collected simultaneously), bivariate Pearson correlations of ≥0.60 are considered ‘adequate’, ≥0.70 for ‘good’, and ≥0.75 for ‘excellent’ correlations. If the correlation is <0.55, the measurements are not measuring the same construct and are thus unrelated.

To estimate internal consistency, we chose McDonald’s coefficient omega (ω), as it is more robust compared to Cronbach’s alpha (α) for deviations from assumptions (item responses should be normally distributed, have equal variance, and explain the construct equally) (e.g., McNeish 2018; Sijtsma 2009). A reliability of 0.70 or higher indicates acceptable internal consistency (Boateng et al. 2018; Taber 2017). Furthermore, we assessed split-half reliability using the odd-even method. This involves grouping odd-numbered items together in one half and even-numbered items in the other. This method is recommended when items are arranged by difficulty level (Schmidt-Atzert and Amelang 2012).

Considering the fairness of the FLUX test, two main approaches are commonly used to test for measurement invariance (or also called measurement equivalence): either DIF (at the item level) within the IRT framework or Multi-Group CFA (MGCFA; to test for factorial structure invariance across groups) (e.g., Hirschfeld and von Brachel 2014; Raju et al. 2002; Reise et al. 1993).

Rasch-based DIF was analysed with facet models carried out using the TAM package (Robitzsch et al. 2022) in R software, version 4.3.0 (R Core Team 2023), to flag items that function differently for different subgroups with the same ability level because of their background characteristics (languages spoken at home, SES, gender) (e.g., AERA et al. 2014). As each item was dichotomously scored, this approach was also preferable over the CFA approach (Raju et al. 2002). Dichotomous SES was determined based on children’s parents’ HISEI scores. Children with scores below the median were classified as low SES, while those with scores equal to or above the median were classified as high SES. Since FLUX is built under Rasch modelling, Paek and Wilson’s (2011) classification rules (or Rasch-based classification rules; for more details, see Paek and Wilson 2011) were used to evaluate DIF instead of the Mantel-Haenszel (Holland and Thayer 1988) procedures, which are more suitable for models developed under classical test theory. The Rasch-based classification rules are based on the difference in item difficulty (γ) between two groups with different background characteristics (natives vs. non-natives; females vs. males; high SES vs. low SES). The rules for classification are as follows: (A) If the value of γ is less than or equal to 0.426, then DIF is considered trivial or negligible. (B) If the value of γ is greater than 0.426 but less than or equal to 0.638, then DIF is considered non-trivial or medium. (C) Finally, if the value of γ is greater than 0.638, DIF is classified as large. In addition, a significance test was performed by dividing the interaction estimate by its standard error to obtain the standardised statistic, known as the z-statistic. Values outside the range of −2 and 2 are considered statistically significant (Wu et al. 2016). In a subtest, DIF items may balance out with an equal number of items favouring each group (Wu et al. 2016). In addition to statistical analysis, it is recommended to provide a reasonable theoretical explanation before omitting items exhibiting DIF (Wu et al. 2016). According to AERA et al. (2014), the presence of DIF does not necessarily signal bias in an item. A suitable and substantial explanation for the DIF is required to conclude that the item is biassed. Removing items without justification is not advisable as it diminishes the test’s psychometric quality and affects the difficulty level (Fleishman et al. 2002; Liu and Rogers 2022). Hence, items exhibiting DIF underwent content analysis by experts.

## 3. Results

### 3.1. Descriptive Statistics

Scale statistics for FLUX, the shortened version of RAVEN’s standard progressive matrices (RAVEN-short), and Educational Achievement (EA) measures are presented in Table A1 (see Appendix A).

Skewness was less than three, and Kurtosis was less than four, showing no severe skew or kurtosis along either measure (see Kline 2011).

### 3.2. Construct and Concurrent Validity

Table 2 summarises scaled CFA statistics by subtest, indicating overall good fit index values for 10 of the 11 subtests.

For subtest QRNS (χ^2^ [44, *N* = 702] = 204.67, *p* < .001, χ^2^/df = 4.65, CFI = 0.85, TLI = 0.810, RMSEA = 0.072, SRMR = 0.089), CFI and TLI were below 0.90, and SRMR was above 0.08, whereas χ^2^/df and RMSEA indicated good fit values.

Three separate CFA were conducted to determine the best fitting factor structure of FLUX (see Figure 2). In these analyses, 132 items are used as observable variables, with each set of items loading on its core subtest. Eleven subtests, their respective four domains (FR, QR, VP, STM), and *Gf* were included in the analysis as factors (latent variables).

As shown in Table 3, the fit index results reveal that all three models fit perfectly with the data. Upon examining the factor loadings of all three models, it was discovered that all items loaded significantly on their relevant core subtest. For Model 1, all subtests loaded significantly on *Gf*, while for Model 2, all subtests loaded significantly on their own respective domain factor (*p* < .001). Considering the hypothesised Model 3, each domain factor also loaded significantly on *Gf* (*p* < .001).

When comparing all three models with the chi-squared difference test (see Table 4), Model 2 and Model 3 were found to best fit the data. While comparing Model 3 with Model 2, analyses reveal that it has a slightly lower fit than Model 2. However, this difference is minimal, especially when measured in such a large sample size.

To further investigate concurrent validity, the FLUX scores were correlated with the scores of the RAVEN-short and EA.

A positive and high correlation of r = 0.71 (N listwise = 648; *p* < .001) with RAVEN-short yields evidence for a good concurrent validity of FLUX. A high correlation was found between RAVEN-short and FLUX’s FR domain (r = 0.72, *p* < .001), as both measure complex figural fluid reasoning.

Criterion validity was examined by correlating FLUX’s total score with EA measures in mathematics (EA-MA), German Listening (EA-GL), and Reading (EA-GR). As shown in Table 5, correlations were highest with EA in mathematics (EA-MA) and lower with EA in German Listening (GL) and Reading (GR). The relatively low correlation (<0.55) with EA-GL and EA-GR yields evidence for divergent validity, which is expected given the language-reduced nature of the FLUX compared to these language-loaded measures. Pearson correlations are presented in Table 5.

### 3.3. Reliability

McDonald’s ω (see Table A1) was acceptable for all four ability domains (QR: ω = 0.79; STM: ω = 0.79; FR: ω = 0.85; VP: ω = 0.87) and the FLUX Full-scale (ω = 0.94). Regarding the subtest level, internal consistency was acceptable for 9 of the 11 subtests (ω = 0.70–0.77), whereas for QRNS (ω = 0.66) and VSYMS (ω = 0.55), McDonald’s ω was below the acceptable value. Spearman–Brown corrected split-half values indicate—except for subtest VSYMS—acceptable to high values (r_tt_ = 0.70 to r_tt_ = 0.95).

### 3.4. Test Fairness

We used DIF detection on the subtest level to ensure fairness in the test. Out of the 132 items, 44 had values of γ below the threshold of 0.426; hence, these items were detected with trivial DIF and classified under group A as negligible.

Twenty-four items had values of γ greater than 0.426 but less than 0.638; hence, these items were detected as non-trivial DIF and were classified under group B as medium. Medium gender-related DIF effects were balanced across the FRA, FRSO, VSR, and CMRT subtests, while a medium SES effect was balanced within the FRA subtest.

Considering medium DIF effects that did not balance out, the distribution extended across all background characteristics. Specifically, some items favoured boys, girls, native speakers, non-native speakers, high-SES, or low-SES children, depending on the subtest.

Only two items exceeded the γ threshold of 0.638. These were classified into Group C (large DIF), but again, their effects were spread across background characteristics and did not consistently favour a single group.

In total, 1.5% of items were flagged for large DIF, and 18.2% were flagged for medium DIF, while 13 out of 132 items displayed unbalanced DIF. However, DIF was distributed across all background characteristics (e.g., three items favouring native speakers and three favouring non-native speakers). Content inspection of these items revealed that they required the same problem-solving strategies or were designed in the same way (for example, using the same colour combination or shape) as items that did not exhibit DIF. Hence, we decided not to exclude items showing DIF, especially because no suitable and substantial explanation for the DIF could be found.

## 4. Discussion

The present study aimed to validate a new tablet-based test battery of cognitive ability designed for multicultural and multilingual primary school environments (grade 3). Standardisation was conducted within Luxembourg’s socioculturally and linguistically heterogeneous educational context. The test includes nonverbal/culture-fair test contents and language-fair instructions presented through child-adapted animated instruction videos. It provides a comprehensive assessment of a child’s general fluid cognitive ability (*Gf*) across four domains: figural fluid reasoning, quantitative fluid reasoning, visual processing, and short-term memory.

CFA statistics indicate empirical support for the hypothesised Model 3 with *Gf* at the highest level, followed by the four cognitive domains at the second level, and 11 subtests at the third level. Hence, *Gf* is found to be a good indicator of a child’s cognitive ability, which aligns with well-known intelligence models (e.g., Cattell 1963; McGrew 2005; Schneider and McGrew 2018; Spearman 1904). Wilhoit’s (2017) proposal that the CHC model can be used to evaluate cognitive abilities in individuals with limited language proficiency has been demonstrated through the selection of non-language-loaded tasks.

According to CFA statistics, Model 2 also has empirical support (without *Gf* at the apex, with subtests loading on their respective cognitive domains). However, as the objective is to develop a test that measures children’s general fluid cognitive ability, Model 3 is preferred over Model 2 in this case. If one wants to evaluate cognitive abilities without assessing general fluid cognitive ability, Model 2—without “*Gf* “ at the apex—can be considered (Schneider and McGrew 2012).

The overall internal consistency of the FLUX Full-scale was relatively high within this sample. Scale statistics at the cognitive domain level showed good to high internal consistency. However, on the subtest level, only 9 out of 11 subtests had acceptable internal consistency. The QRNS (Quantitative Reasoning—Numerical Series) subtest from the domain Quantitative fluid reasoning (QR) and the VSYMS (Visual Symbolic Memory Span) subtest from the domain Short-term memory (STM) had low internal consistency. Thus, in the case of highly specific diagnostic questions, when one decides to assess individual subtests, it is recommended to assess those subtests together with subtests measuring the same cognitive domain and never alone (e.g., QRNS with Non-Symbolic Quantitative Reasoning [NQR] to assess QR and never alone). To enhance future test reliability, especially for the two subtests, low-correlating items should be replaced with better, new ones that demonstrate a higher correlation with the respective subscale. To achieve this, the newly developed items should undergo a pilot and pre-test phase again before being incorporated into the test.

Correlations with the nonverbal RAVEN-short test (Langener et al. 2021) and Educational Achievement measures provided support for FLUX’s concurrent and criterion-related validity. These findings suggest that FLUX is measuring what it is intended to measure—namely, general fluid cognitive ability. As RAVEN-short assesses complex figural reasoning through abstract figures, it shows a high correlation with FLUX’s figural fluid reasoning domain, reflecting their shared emphasis on abstract reasoning processes.

FLUX correlated higher with the Educational Achievement measure in EA-MA than with highly language-loaded measures, providing support for its convergent validity. Its low correlation with high language-loaded measures, EA-GR and especially EA-GL, suggests that FLUX is not a language-dominant test and does not assess the same construct as EA measures in GR and GL, yielding evidence for divergent validity. Moreover, one should keep the heterogeneity of the multilingual sample in mind, as it may have contributed to the lower correlation value, as 60.2% of the children in the sample do not speak Luxembourgish and/or German at home, which may have resulted in lower performance on language-loaded EA measures while performing higher on the language-fair FLUX test.

Another explanation might be the age of the target group. Children’s language abilities in German may not be as developed (crystallised) as their mathematical abilities, as the majority of children only start to acquire German language skills in grade 1 when formal schooling starts (MENJE 2020), whereas they may have already been exposed to less language-loaded mathematical concepts through their parental education or early exposure in kindergarten. This pattern aligns with Cattell’s (1963) claim that cognitive ability depends on the current state of brain development and neural processes observed through *Gf*, which is innate and contributes through learning to the formation of *Gc* (knowledge gained via parental or school education) (Cattell 1963; Schweizer and Koch 2002).

Out of the 132 items, 1.5% were flagged for large DIF, while 18.2% were flagged for medium DIF. Content inspection by a group of experts revealed no suitable and substantial explanation for the DIF of these items. As per the recommendation of AERA et al. (2014), these items were thus not removed. Also, according to Liu and Rogers (2022), deleting DIF items without cause is not recommended since it reduces reliability, content validity, and the number of items with a specific level of difficulty (Fleishman et al. 2002; Liu and Rogers 2022).

IRT was used to assess the psychometric properties of items, including discrimination and difficulty. The availability of a variety of difficulty levels might allow for the identification of intellectual giftedness (high cognitive potential) and intellectual disability (low cognitive potential). Therefore, together with measures of educational achievement or school grades, FLUX could also be used to detect children who are either underachievers or overachievers in school. However, since the main aim of this study was to develop a culture and language-fair test battery, standardisation had not yet been extended to children formally diagnosed with intellectual giftedness or disability. Although the standardisation sample may include undiagnosed children from these groups, specific norming studies are necessary to establish appropriate benchmarks and to include more challenging or easier items, ensuring accurate assessment across the full ability range.

Overall, the psychometric properties enumerated above indicate that the test can be considered an appropriate assessment tool for evaluating *Gf* in multicultural and multilingual children. Further research is necessary to investigate the test’s retest reliability, which can be achieved by assessing them twice within a short time frame and correlating children’s results of both assessments. To be able to do this, future research could adapt FLUX to a wider range of age groups to allow a broader field of application and, thus, also pave the way for longitudinal data. This would also allow for demonstrating the predictive validity of the test regarding academic performance, which is an important aspect of test evaluation. However, this could not be examined in the present study, as the design did not allow for follow-up data collection. In case of underachievement, future longitudinal research could also explore if early identification of underachievers and providing support could minimise underachievement and promote academic success.

The cognitive test battery was developed specifically for Luxembourg’s multilingual school system, where students communicate in three official languages in their classrooms and their native language at home. This can be considered the perfect context for designing a test that meets the unique requirements of multilingual educational settings. Such tests are increasingly important in today’s world due to the rising number of multicultural and multilingual classrooms caused by global migration and mobility (Parveen et al. 2022).

The test should be applicable in other multicultural and multilingual educational settings. Nevertheless, although the standardisation sample in the present study was socioculturally and linguistically heterogeneous, it may still differ in some characteristics from other populations. Future research should therefore further examine test fairness and establish the cross-cultural validity of the FLUX test by replicating findings across both Western and non-Western contexts. The latter may be less familiar with such standardised testing formats to assess cognitive abilities and place greater importance on motivation and social skills (Neisser et al. 1996), which could, in turn, influence test validity.

The FLUX test provides assessments of multiple domains, which allows the creation of a child’s profile, identifying their strengths and weaknesses (Wilhoit 2017). The manual has therefore foreseen this by including critical difference values to determine whether observed score variations reflect significant differences in ability or result from measurement error resulting from natural fluctuations, due to external factors such as noise or tiredness. This information is crucial for helping to develop personalised learning plans and interventions as early as possible. Moreover, recognising that all psychological measures are subject to some degree of measurement error, results will be reported within a 90% confidence interval, indicating the range within which the true score is expected to lie.

Furthermore, FLUX has been found to effectively evaluate a child’s cognitive ability as a group power test, which also allows for an economic assessment (e.g., Ford and Dahinten 2005). Even though it is standardised as a group test, it allows children to receive individual feedback during the training phase and work individually at their own pace, thereby helping to ensure a more culturally fair context (e.g., Kim and Zabelina 2015) and a reliable assessment of children’s performance since it was administered on a tablet (Lavergne and Vigneau 1997).

However, it is important to determine whether the results of group-based norms are comparable with the results of individual testing. In practice, group-based norms are consistently applied to individual testing (Baudson and Preckel 2013). As the norms are group-based, it is advisable to supplement the test with other measurements if used in decisive educational or diagnostic settings. Factors such as personality, social skills, creativity, and motivation are also crucial in shaping one’s overall cognitive abilities or potential and should not be ignored.

In addition, although the test was designed as a power test, allowing children enough time to respond to each item, including a control condition with a speed-test version of FLUX that incorporates reaction time into its scoring, would have been valuable to further confirm its cultural fairness.

Using FLUX as a tablet-based assessment appears feasible. This is in line with Pitchford and Outhwaite’s (2016) finding that touch-screen tablet technology can provide reliable and valid psychometric measures in the school context. Hence, tablet technology shows promise for cross-cultural comparisons and research. Moreover, the use of tablets was found to be highly engaging for children. The test materials are child-friendly, colourful, and age-appropriate, which may help alleviate anxiety and maintain motivation during the testing process. Colour combinations were also carefully selected, in close collaboration with experts in vision, to ensure accessibility for children with visuo-perceptual deficiencies, such as colour blindness.

As materials can be easily adapted to tactile materials, future research could adapt the test to totally visually impaired children for clinical validation. Clinical validation samples may also include children with hearing disability, especially because video animations have already been adapted to also be understood without sound. Additionally, children with learning disorders can be assessed in an adapted manner as well. As FLUX was designed to be language-fair, there is no verbal and/or language-based stimuli, allowing for children with learning disorders in reading and/or writing to be assessed without any adaptation. For children with learning disorders in mathematics, the standardisation of the test and final development of the manual has foreseen the possibility to assess cognitive ability without the second cognitive domain QR—which is the only domain with numerical stimuli—and to still receive a valid score.

## 5. Conclusions

To conclude, the present study provides evidence that FLUX can be considered an accurate, multidimensional and fair measure of a child’s cognitive ability. These findings suggest that FLUX could serve as a promising alternative to traditional tests that are paper-and-pencil administered and/or use verbal test instructions.

These findings are encouraging as they represent a crucial step towards promoting equal opportunities in a very heterogeneous multilingual educational context, additionally accounting for children with specific needs (such as visual deficiencies). By reducing the cultural and linguistic demands inherent in many assessments, FLUX not only minimises the risk of disadvantaging children with limited proficiency in the language of instruction but may also contribute to more equitable and valid diagnostic practices.

## Figures and Tables

**Table 1:** Sample Characteristics.

	N	Percentage	Mean	SD
Age (years)	-	-	8.85	0.66
Gender	703	100	-	-
Female	343	48.8	-	-
Male	359	51.1	-	-
No Information	1	0.1	-	-
Language	703	100		
Native speakers	279	39.7	-	-
Non-native speakers	423	60.2	-	-
No Information	1	0.1	-	-
SES (HISEI)	568	100	48.76	16.87
High	286	50.4	63.91	5.89
Low	282	49.6	33.39	8.26

**Table 2:** Fit Index Values for Tested Models per FLUX subtest.

First-Order Models	χ^2^	df	χ^2^/df	CFI	TLI	RMSEA	SRMR
FRM	146.45	65	2.25	0.953	0.944	0.042	0.066
FRA	140.46	65	2.16	0.942	0.930	0.041	0.079
FRSO	121.58	65	1.87	0.966	0.959	0.036	0.066
QRNS	204.67	44	4.65	**0.848**	**0.810**	0.072	**0.089**
NQR	228.45	90	2.54	0.934	0.923	0.047	0.069
FRR	110.63	54	2.05	0.971	0.965	0.040	0.057
VSR	41.74	35	1.19	0.995	0.994	0.017	0.059
PFR	168.20	65	2.59	0.930	0.916	0.049	0.072
CMRT	74.31	35	2.12	0.976	0.970	0.040	0.065
VSYM	53.83	35	1.54	0.951	0.937	0.028	0.064
VSM	63.02	54	1.17	0.995	0.994	0.015	0.050

*Note.* **χ**^2^ = Chi-Square value, df = degree of freedom, CFI: Comparative Fit Index, RMSEA: Root Mean Square Error of Approximation, TLI: Tucker–Lewis Index, SRMR: Standardised Root Mean Square Residual; All fit index values with a bad fit are in **bold**.

**Table 3:** Robust Test Statistics; fit Index Values of Tested Models.

Models	χ^2^	df	χ^2^/df	CFI	TLI	RMSEA (90% CI)	SRMR
Model 1	9202.06	8503	1.08	0.962	0.962	0.011 (0.009–0.013)	0.069
Model 2	9068.05	8497	1.07	0.969	0.969	0.010 (0.008–0.012)	0.067
Model 3	9081.33	8499	1.07	0.968	0.968	0.010 (0.008–0.012)	0.067

*Note.***χ**^2^ = Chi-Square value, df = degree of freedom, CFI: Comparative Fit Index, RMSEA: Root Mean Square Error of Approximation, TLI: Tucker–Lewis Index, SRMR: Standardised Root Mean Square Residual, CI = Confidence Interval.

**Table 4:** Standard Test Statistics: Comparing Tested Models With the Chi-square Difference Test.

Model	df	χ^2^	Δχ^2^	Δdf	CFI	TLI	RMSEA (90% CI)	SRMR
Model 2 vs.	8497	8889.6	-	-	0.995	0.995	0.008 (0.006–0.011)	0.067
Model 1	8503	9322.2	88.023 ***	6	0.990	0.989	0.012 (0.010–0.013)	0.069
Model 3	8499	8935.9	9.6971 **	2	0.994	0.994	0.009 (0.006–0.011)	0.067
Model 3 vs.	8499	8935.9	-	-	0.994	0.994	0.009 (0.006–0.011)	0.067
Model 1	8503	9322.2	79.339 ***	4	0.990	0.989	0.012 (0.010–0.013)	0.069

*Note.* ** *p* ≤ .01; *** *p* < .001, χ^2^ = Chi-Square value, df = degree of freedom, Δχ^2^ = Chi-Square difference value, Δdf = difference value of degrees of freedom, CFI: Comparative Fit Index, RMSEA: Root Mean Square Error of Approximation, TLI: Tucker–Lewis Index, SRMR: Standardised Root Mean Square Residual, CI = Confidence Interval.

**Table 5:** Correlations Between FLUX and Educational Achievement (EA) Measures in Mathematics (MA), German Listening (GL) and German Reading (GR).

		*N* (Listwise) = 586		
		Educational Achievement		
		EA-MA	EA-GL	EA-GR
Domain level	FR	0.546 **	0.220 **	0.318 **
	QR	0.487 **	0.141 **	0.246 **
	VP	0.527 **	0.228 **	0.303 **
	STM	0.449 **	0.103 *	0.247 **
Full-scale	FLUX	0.617 **	0.220 **	0.345 **
	RAVEN-Short	0.496 **	0.194 **	0.278 **

*Note.* * *p* ≤ .05; ** *p* < .01, FR = Figural reasoning, QR = Quantitative reasoning, VP = Visual processing, STM = Short-term memory, EA = Educational Achievement measures in: -MA = Mathematics, -GL = German Listening, -GR = German Reading.

## Data Availability

The data presented in this study are available on request from the corresponding author. The data analysed in our study is sensitive cognitive data. The data has been anonymised for the purpose of research to develop and validate a norm-referenced tablet-based test battery called FLUX (Fluid Intelligence Luxembourg), tailored to the specific needs of multilingual educational settings. Hence, it is not possible to identify any child, however we do not have permission to share this highly sensitive data online.

## Appendix A

**Table A1:** Scale Statistics for FLUX, RAVEN-Short and Educational Achievement Measures.

Levels	Scale	N (Listwise)	Number of Test Items	Mean	SD	Variance	McDonald’s ω	r_tt_	Skewness (SE)	Kurtosis (SE)
	FRM	703	13	7.53	2.87	8.26	0.71	0.70	−0.43 (0.09)	−0.44 (0.18)
	FRA	703	13	6.90	2.69	7.25	0.70	0.70	0.03 (0.09)	−0.65 (0.18)
	FRSO	672	13	8.12	2.82	7.92	0.73	0.75	−0.26 (0.09)	−0.72 (0.19)
	QRNS	702	11	5.71	2.56	6.54	0.66	0.70	0.08 (0.09)	−0.66 (0.18)
Subtest level	NQR	701	15	8.68	3.39	11.51	0.75	0.79	−0.03 (0.09)	−0.90 (0.18)
	FRR	672	12	5.57	3.12	9.73	0.77	0.80	0.42 (0.09)	−0.80 (0.19)
	VSR	659	10	4.51	2.34	5.48	0.75	0.76	0.33 (0.10)	−0.58 (0.19)
	PFR	670	13	6.77	2.97	8.85	0.72	0.73	0.24 (0.09)	−0.71 (0.19)
	CMRT	702	10	5.08	2.42	5.86	0.75	0.77	−0.16 (0.09)	−0.61 (0.18)
	VSYMS	672	10	5.00	1.96	3.82	0.55	0.55	−0.08 (0.09)	−0.44 (0.19)
	VSM	701	12	5.74	2.76	7.64	0.75	0.73	0.23 (0.09)	−0.72 (0.18)
	FR	672	39	22.57	6.93	47.99	0.85	0.85	−0.13 (0.09)	−0.70 (0.19)
	QR	700	26	14.39	4.98	24.83	0.79	0.84	0.07 (0.09)	−0.65 (0.19)
Domain level	VP	653	35	16.91	7.01	49.07	0.87	0.90	0.49 (0.10)	−0.52 (0.19)
	STM	669	32	15.79	5.24	27.45	0.79	0.82	−0.02 (0.09)	−0.45 (0.19)
Full-scale	FLUX	648	132	69.83	19.90	395.64	0.94	0.95	0.19 (0.10)	−0.65 (0.19)
	RAVEN-Short	702	15	7.76	3.36	11.31	0.78	0.80	−0.17 (0.09)	−0.65 (0.18)
	EA-MA	655	-	477.89	115.29	13292.18	-	-	0.24 (0.10)	0.90 (0.19)
Educational Achievement	EA-GL	631	-	469.91	105.04	11033.20	-	-	0.27 (0.10)	−0.47 (0.19)
	EA-GR	630	-	474.02	127.01	16131.44	-	-	0.21 (0.10)	−0.36 (0.19)

*Note.* FRM = Figural Reasoning—Matrices, FRA = Figural Reasoning—Analogies, FRSO = Figural Reasoning—Sequential Order, QRNS = Quantitative Reasoning—Numerical Series, NQR = Non-Symbolic Quantitative Reasoning, FRR = Figural Rotation Reasoning, VSR = Visual Spatial Reasoning, PFR = Paper Folding Reasoning, CMRT = Counting-Memory-Recall Task, VSYMS = Visual Symbolic Memory Span, VSM = Visual-Spatial Memory, FR = Figural reasoning, QR = Quantitative reasoning, VP = Visual Processing, STM = Short-term memory, EA = Educational Achievement measures in: -MA = Mathematics, -GL = German Listening, -GR = German Reading, SD = Standard Deviation.
